# Systematic review of donor and recipient predictive biomarkers of response to faecal microbiota transplantation in patients with ulcerative colitis

**DOI:** 10.1016/j.ebiom.2022.104088

**Published:** 2022-05-31

**Authors:** Nia Paddison Rees, Walaa Shaheen, Christopher Quince, Chris Tselepis, Richard D Horniblow, Naveen Sharma, Andrew D Beggs, Tariq H Iqbal, Mohammed Nabil Quraishi

**Affiliations:** a University of Birmingham Microbiome Treatment Centre, Birmingham, UK; b Institute of Cancer and Genomic Sciences, University of Birmingham, UK; c Earlham Institute, Norwich, UK; d Institute of Clinical Sciences, School of Biomedical Sciences, University of Birmingham, UK; e University Hospitals Birmingham NHS Foundation Trust, Birmingham, UK; f Institute of Microbiology and Infection, University of Birmingham, UK

**Keywords:** Ulcerative colitis, faecal microbiota transplantation, biomarkers

## Abstract

**Background:**

Faecal microbiota transplantation (FMT) has previously been explored as a treatment for ulcerative colitis (UC) however, biomarkers that predict and / or are associated with clinical response are poorly defined. The aim of this systematic review was to identify donor and recipient clinical, microbial and metabolomic predictive biomarkers of response to FMT in UC.

**Methods:**

A systematic search of the relevant literature of studies exploring FMT in UC was conducted. Data on microbial diversity, taxonomic changes, metabolic changes, donor and recipient microbiota relationship and baseline predictors was examined.

**Findings:**

2852 studies were screened, and 25 papers were included in this systematic review. Following FMT, alpha diversity was seen to increase in responders along with increases in the abundance of *Clostridiales* clusters (order) and *Bacteroides* genus. Metabolomic analysis revealed short chain fatty acid (SCFA) production as a marker of FMT success. Donors or FMT batches with higher microbial alpha diversity and a greater abundance of taxa belonging to certain Bacteroides and *Clostridia* clusters were associated with clinical response to FMT. Baseline clinical predictors of response in patients with UC included younger age, less severe disease and possibly shorter disease duration. Baseline recipient microbial predictors at response consisted of higher faecal species richness, greater abundance of *Candida* and donor microbial profile similarity.

**Interpretation:**

Distinct changes in gut microbiota profiles post-FMT indicate that certain baseline characteristics along with specific microbial and metabolomic alterations may predispose patients towards a successful therapeutic outcome. Opportunities towards a biomarker led precision medicine approach with FMT should be explored in future clinical studies.

**Funding:**

There no specific funding to declare.

**Research in context**

**Evidence before this study**

To date, eight double-blind randomised placebo-controlled trials on the use of FMT to treat UC have been published, 6 of which have reported positive findings. Whilst these studies highlight the capability of FMT to ameliorate UC, very little is known about the underpinning mechanisms. The lack of well-defined biomarkers and treatment targets makes it pragmatically challenging to determine the frequency and interval at which treatment with FMT should be administered.

**Added value of this study**

Through a systematic review of the current evidence base, we describe clinical, microbial and metabolomic biomarkers that are predictive of response at baseline (pre-FMT), and are associated with response following FMT treatment in patients with active UC.

**Implications of all the available evidence**

The findings of this systematic review highlight the possibility of enhancing a sustained response to FMT through biomarker-based selection and optimisation of donors and patients before and during the treatment with FMT. Utilising precision medicine in this field deserves further exploration as it has the potential to facilitate an individualised, biomarker driven ‘treat to microbiome/metabolome’ target approach with FMT in patients with UC.

Alt-text: Unlabelled box

## Introduction

Ulcerative colitis (UC) is a subtype of inflammatory bowel disease (IBD) that is characterised by chronic inflammation of the colonic mucosa with patients typically presenting with bloody diarrhoea.^1^ Whilst the precise aetiology of UC remains unclear, it is considered to be triggered by dysregulated and sustained immune responses to gut microbiota in genetically susceptible individuals.^2^^,^^3^ Patients with UC possess an altered gut microbiota composition, known as dysbiosis, characterised by reduced microbiota diversity, decrease in the phylum Bacteroidetes and Firmicutes along with a corresponding increase in Proteobacteria.^4^^,^^5^ This has led to a focus on the modulation of gut bacteria as a treatment method for UC, primarily through faecal microbiota transplantation (FMT). FMT is the procedure of transferring processed faecal matter of a healthy individual into another individual with a microbiota mediated disease.^6^

To date, eight double-blind randomised placebo-controlled trials (RCTs) on the use of FMT to treat UC have been published, 6 of which have reported positive findings.^7^, ^8^, ^9^, ^10^, ^11^, ^12^, ^13^, ^14^ Whilst these studies highlight the capability of FMT to ameliorate UC, very little is known about the underpinning mechanisms. The heterogeneity in study designs both with regards to FMT preparation and administration protocols as well as patient selection makes it challenging to draw solid conclusions for its adoption into clinical practice. Furthermore, it remains unclear if specific donor or recipient characteristics may predict response to FMT or denote successful response following FMT.^15^^,^^16^ This lack of well-defined biomarkers and treatment targets makes it pragmatically challenging to determine the frequency and interval at which treatment with FMT should be administered.

Currently, there are no published systematic reviews that explore predictive biomarkers of FMT in patients with UC. This systematic review aims to answer whether clinical, microbial and metabolomic predictive biomarkers exist and if so, which of these are predictive of response at baseline (pre-FMT), and are associated with response following FMT treatment in patients with active UC.

## Methods

### Search strategy and study selection

The systematic review was conducted in accordance with preferred reporting items for systematic reviews and meta-analyses (PRISMA) criteria. The databases MEDLINE, EMBASE, CINAHL and Cochrane Library, were searched for suitable articles from commencement to January 2022 using search terms outlined (Supplement Table 1). In addition, references included in earlier review articles were searched to identify any additional studies. Results from the searches were imported into a bibliography manager (EndNote X9) and duplicate studies were removed.

**Table 1:** Randomised control studies of FMT in ulcerative colitis

Reference	No of subjects	Control/ Comparator	Treatment	Median / Mean Age(years)	Gender(% Male)	Average disease severity indices at baseline	Treatment Duration	Relevant study characteristics
Paramsothy et al* (2019)^33^	81	Placebo group (n = 40)	**Treatment protocol** Initial colonoscopic infusion followed by intensive FMT infusion enemas (n = 41) **FMT preparation** Pooled from multiple donors	FMT arm - 35.6 (27.8-48.9) Placebo arm - 35.4 (27.7-45.6)	FMT arm – 54% Placebo arm – 63%	FMT arm – 8 (average Total Mayo score) Placebo arm – 8 (average Total Mayo score)	FMT treatment 5 days/week for 8 weeks	Patients in the placebo group were eligible to receive open-label FMT after the double-blind study period 314 faecal samples collected from the patients at screening, every 4 weeks during treatment, and 8 weeks after the blinded or open-label FMT therapy
Moayyedi *et al* (2015)^8^	75	Placebo group (n = 37)	**Treatment protocol** Examined by flexible sigmoidoscopy followed by FMT infusion via enema (n = 38) **FMT preparation** Single donor per patient	FMT arm – 42.2 (±15.0) Placebo arm – 35.8 (±12.1)	FMT arm – 47% Placebo arm – 70%	FMT arm – 8.24 (±2.61) Total Mayo Clinic score Placebo arm – 7.86 (±2.28) Total Mayo Clinic score	FMT treatment 1 day/week for 6 weeks	Patients provided stool samples when the study began and during each week of FMT for microbiome analysis
Costello *et al* (2019)^9^	73	Autologous FMT control group (n = 35)	**Treatment protocol** Anaerobically prepared pooled donor FMT via colonoscopy followed by 2 enemas over 7 days (n = 38) **FMT preparation** Pooled from multiple donors	Donor FMT arm – 38.5^28^, ^29^, ^30^, ^31^, ^32^, ^33^, ^34^, ^35^, ^36^, ^37^, ^38^, ^39^, ^40^, ^41^, ^42^, ^43^, ^44^, ^45^, ^46^, ^47^, ^48^, ^49^, ^50^, ^51^, ^52^ Autologous FMT arm – 35^25^, ^26^, ^27^, ^28^, ^29^, ^30^, ^31^, ^32^, ^33^, ^34^, ^35^, ^36^, ^37^, ^38^, ^39^, ^40^, ^41^, ^42^, ^43^, ^44^, ^45^, ^46^	dFMT– 53% aFMT – 57%	dFMT arm – 7.2 (±1.7) Mean Total Mayo score aFMT – 7.4 (±1.9) Mean Total Mayo score	FMT treatment per week with patients monitored at 8 weeks and 12 months post-FMT	Open-label therapy was offered to autologous FMT participants at 8 weeks and they were followed up for 12 months Recipient stool samples were collected at baseline (week 0) and weeks 4, 8, and 52 for microbiome, metabolome, and faecal calprotectin assessment
Rossen *et al* (2015) †^10^	48	Autologous FMT control group (n = 25)	**Treatment protocol** Pre-treatment with bowel lavage followed by 2 duodenal infusions of a suspension of donor faeces via nasoduodenal tube (n = 23) **FMT preparation** Single donor per patient	Donor FMT arm – 40^33^^-56^ Autologous FMT arm – 41^30^, ^31^, ^32^, ^33^, ^34^, ^35^, ^36^, ^37^, ^38^, ^39^, ^40^, ^41^, ^42^, ^43^, ^44^, ^45^, ^46^, ^47^, ^48^	dFMT arm – 47.8% aFMT arm – 44%	dFMT arm – 10^5^, ^6^, ^7^, ^8^, ^9^, ^10^, ^11^ Median SCCAI score aFMT arm – 8^4^, ^5^, ^6^, ^7^, ^8^, ^9^, ^10^, ^11^ Median SCCAI score	FMT treatment at the start of the study (week 0) and 3 weeks later (week 3)	Faecal samples were collected at baseline before bowel lavage and 6 and 12 weeks after FMT
Crothers *et al* (2021)^13^	12	Placebo group (n = 6)	**Treatment protocol** FMT induction by colonoscopy, followed by oral administration of frozen encapsulated cFMT (n = 6) **FMT preparation** Single donor for induction Multiple (2 pre-defined) donors during maintanence	FMT arm – 41 (±15) Placebo arm – 52 ±15)	FMT arm - 67% Placebo arm - 50%	FMT arm – 6.3 (±2.0) Mean Total Mayo score Placebo arm – 6.7 (±1.2) Mean Total Mayo score	Daily cFMT treatment for 12 weeks	Subjects were followed for 36 weeks and longitudinal clinical assessments Subjects in both arms of the study were pre-treated with antibiotics for 7 days prior to FMT (or placebo) procedure Subject stool samples were obtained weekly throughout the study period, beginning prior to antibiotic pre-treatment, and ending at 18-weeks follow-up
Pai *et al* (2021)^12^	25	Placebo group (n = 12)	**Treatment protocol** FMT administered by rectal enema (n=13) **FMT preparation** Multiple donors per patient (not pooled)	Overall 10.5^4^, ^5^, ^6^, ^7^, ^8^, ^9^, ^10^, ^11^, ^12^, ^13^, ^14^, ^15^, ^16^, ^17^ Individual arms not specified	Not specified	Not specified	Total 12 enemas (given biweekly)	Seven patients randomized to the placebo arm crossed over to the open-label arm after 30 weeks of placebo treatment
Haifer *et al* (2021)^11^	35	Placebo group (n=20)	**Treatment protocol** Six FMT capsules four times a day for 1 week, then six capsules twice daily for 1 week, followed by six capsules daily for the remaining 6 weeks. Each capsule contains 0.35g lyophilised stool. (n=15) **FMT preparation** Two donors, unclear if pooled	FMT arm - 37.1 (31.8–46.8) Placebo arm - 36.7 (25.1–42.0)	FMT arm – 60% Placebo arm – 45%	FMT arm - 5^5^, ^6^, ^7^, ^8^, ^9^ median total Mayo score Placebo arm - 7^5^, ^6^, ^7^, ^8^ median total Mayo core	8 weeks of capsules during induction, followed by 2 capsules daily for remaining 58 weeks for maintenance.	Antibiotic pre-treatment in both groups. 10 patients randomised to FMT arm with clinical response entered maintenance phase of the study - 4 assigned to FMT and 6 assigned to FMT withdrawal

⁎ Further post hoc microbiome and mycobiome analysis reported separately^32^^,^^33^

† Further post hoc microbiota analysis reported separately^31^ FMT-faecal microbiota transplantation, cFMT-capsulised faecal microbiota transplantation, dFMT-donor FMT, aFMT-autologous FMT, SCCAI-simple clinical colitis activity index.

Randomised control trials (RCTs) and non-randomised studies were included with exclusion of case reports and conference abstracts. Double blind RCTs were further split based on comparators (placebo and non-placebo controlled studies). Studies consisting of patients of all ages with active UC examining any of the following: clinical, microbial (diversity and taxonomic changes) and metabolomic biomarkers at baseline and post FMT treatment predictive of induction and maintenance of clinical remission in patients with active UC were included. Studies were excluded if they had under 10 patients in the FMT treatment arm or only included patients with concurrent infections. No restriction on language or the comparator type for comparative study designs was implemented. Abstracts of the papers identified by the initial search were evaluated by the lead and senior authors for appropriateness to the study question. All relevant papers were obtained and analysed in detail. Articles were independently assessed by two reviewers using pre-defined eligibility criteria and any disagreements were resolved by consensus.

### Data extraction

Data was extracted independently by the two reviewers onto a Microsoft Excel spreadsheet (Microsoft, Washington, USA) from the eligible studies. Data relating to donor and patient demographics, treatment groups/comparator(s) and outcome measures were collected. Exploratory data on changes in alpha and beta diversity, microbial taxa, metabolome and donor-patient microbiota similarities following FMT were collected. No unclear or missing data was noticed which would have required approaching the study authors for clarification. Risk of bias of the included RCTs was assessed with the Cochrane Collaboration's risk of bias tool and non-randomised/cohort studies was with the Newcastle-Ottawa quality assessment scale (NOS).^17^^,^^18^ If there were any discrepancies a third reviewer was consulted.

### Role of funders

No specific funding has been received for this systematic review. This is independent work conducted by the authors of the review.

## Results

### Study characteristics

**Figure 1:**
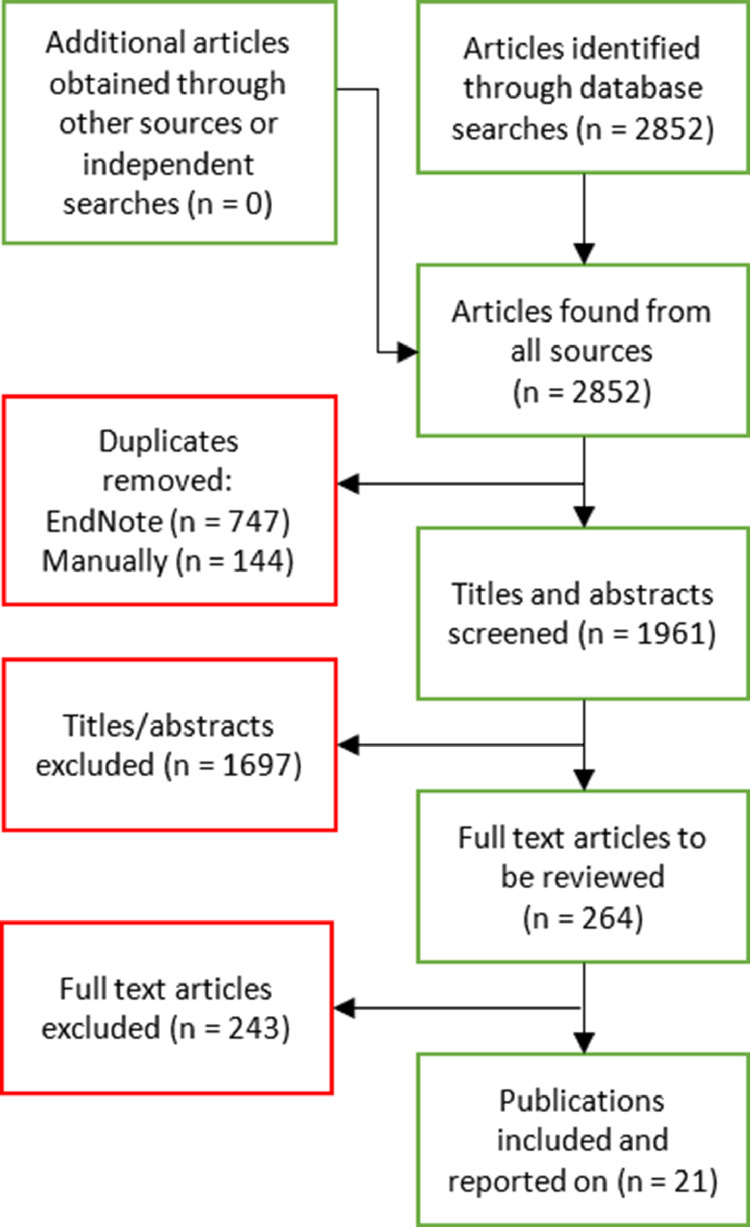
PRISMA flow diagram summarising the screening process for the systematic review

The search strategy generated 2852 citations from which 25 articles investigating the use of FMT in UC patients satisfied the study selection criteria for further assessment (Figure 1). Of these, 7 were placebo controlled double blind RCTs^7^, ^8^, ^9^, ^10^, ^11^, ^12^, ^13^ (Table 1; total of 8 RCTs but one did not report predictive associations and failed to meet inclusion criteria for this systematic review), 2 were non-placebo controlled blinded randomised studies^19^^,^^20^ and 14 were non-randomised or observational studies^21^, ^22^, ^23^, ^24^, ^25^, ^26^, ^27^, ^28^, ^29^, ^30^ (Table 2). In addition, 2 studies performed post-hoc microbiota analysis from their placebo controlled double blind RCTs.^31^^,^^32^ All the RCTs received a low bias ranking overall (Supplementary Table 2). None of the non-randomised / cohort studies scored at the highest end of the NOS scale, with a mean score of 5 (range 4 to 6) out of 9 (Supplementary Table 3).

**Table 2:** Non-placebo controlled blinded randomised trials and non-randomised studies of FMT in ulcerative colitis*

Reference	No. of Subjects	Control/Comparator	Treatment	Median / Mean Age (years)	Gender (% Male)	Average disease severity indices at baseline	Treatment duration	Relevant study characteristics
Brezina *et al* (2021) †^20^	45	5-ASA treatment group (n = 22)	**Treatment protocol** Multi-session FMT enemas (n = 23) **FMT preparation** Single donor per patient	FMT arm – 39^25^^-63^ 5-ASA arm – 39.5^27^^-70^	FMT arm – 52% 5-ASA arm – 50%	FMT arm – 6^4^, ^5^, ^6^, ^7^, ^8^, ^9^, ^10^ Total Mayo score 5-ASA arm – 6^4^, ^5^, ^6^, ^7^, ^8^, ^9^, ^10^ Total Mayo score	FMT treatment 5 times in the first week and then once weekly for 5 weeks	Faecal samples were collected at baseline and each study visit at weeks 2, 4, 6, and 12 in the FMT group and at the 1 year follow-up in all patients
Shabat *et al* (2021) †^19^	51	Single donor FMT by colonoscopy without dietary conditioning of donors (group 1 (n = 17)), UC Exclusion Diet (UCED) alone for patients (group 3 (n = 15))	**Treatment protocol** FMT by colonoscopy with dietary pre-conditioning of the donor for 14 days and a UCED for the patients (group 2 (n = 19)) **FMT preparation** Single donor per patient	Group 1 – 43.1 (±14.7) Group 2 – 43.5 (±10.5) Group 3 – 33.3 (±9.8)	Group 1 – 70.6% Group 2 – 73.7% Group 3 – 73.3%	Group 1 – 7^6^, ^7^, ^8^, ^9^ Median SSCAI score Group 2 – 8^6^, ^7^, ^8^, ^9^, ^10^ Median SCCAI score Group 3 – 6^5^, ^6^, ^7^, ^8^ Median SCCAI score	FMT by colonoscopy on day 1 and rectal enemas on days 2 and 14	Three arm study exploring role of donor and recipient dietary conditioning in optimisation of response to FMT
Tian *et al* (2019)^21^	20	Before vs after FMT treatment (+ comparison against the healthy donor profile)	**Treatment protocol** Pre-treatment with bowel lavage followed by FMT via gastroscopy **FMT preparation** Single donor per patient	62.50 (±77.14)	55%	5.00 (±2.75) Mayo score	FMT treatment 5 times, once every 3 weeks	16S ribosomal RNA sequencing analysis performed on the bacterial rRNA from stool of healthy donors and patients with UC before treatment and after the first and second treatment (groups d0, d1, and d2)
Li *et al* (2020)^22^	202	Before vs after FMT treatment (+ comparison against the healthy donor profile)	**Treatment protocol** Single FMT via gastroscopy **FMT preparation** Single donor per patient	36 (29-49.25)	58.2%	7^5^, ^6^, ^7^, ^8^, ^9^ Median Partial Mayo score	Patients received 1 infusion (2^nd^ and 3^rd^ courses were given to patients who relapsed)	42 UC stool samples were included: 22 samples at baseline and 20 at 5 days after the first course of FMT
Kump *et al* (2018)^23^	27	Antibiotic pre-treatment only group (n = 10)	**Treatment protocol** Multi-session FMT via colonoscopy / flexible sigmoidoscopy (n = 17) **FMT preparation** Single donor per patient	FMT arm – 44 (±18) Antibiotic arm – 36 (±13)	FMT arm - 82% Antibiotic arm – 30%	FMT arm – 8.9 (±1.6) Mean Total Mayo score Antibiotic arm – 8.1 (±3.1) Mean Total Mayo score	FMT treatment 5 times, at 14-day intervals (1^st^ treatment given via ileocolonoscopy, the 4 subsequent sessions at days 14, 28, 42 and 56 were via flexible sigmoidoscopy)	Faecal samples for microbiota analyses were collected at each study visit
Jacob *et al* (2017)^24^	20	Before vs after FMT treatment (+ comparison against the healthy donor profile)	**Treatment protocol** Single FMT delivery via colonoscopy **FMT preparation** Pooled from multiple donors	38.4 (±12.6)	60%	8.1 (±2,4) Mean Total Mayo score	Patients received 1 infusion	16S rRNA gene sequencing was performed on recipient faecal DNA samples pre- and 2 and 4 weeks post- transplant
Fang *et al* (2021)^25^	20	FMT routine therapy control group (n = 10)	**Treatment protocol** Monotherapy with a single fresh FMT via colonoscopy (n = 10) **FMT preparation** Single donor per patient	FMT arm – 51.5 (±12.7) Control arm – 44.6 (±14.9)	Unclear	FMT arm – 9.5 (±2.5) Total Mayo score Control arm – 8.6 (±2.9) Total Mayo score	Patients received 1 infusion	Fresh faecal samples from the donors and pre-FMT and post-FMT samples from patients were collected
Cui *et al* (2015)^26^	15	Before vs after FMT treatment (+ comparison against the healthy donor profile)	**Treatment protocol** Step-up FMT treatment via endoscopic infusion tube **FMT preparation** Unclear if multiple / pooled donors or single donor per patient	31.7^11^, ^12^, ^13^, ^14^, ^15^, ^16^, ^17^, ^18^, ^19^, ^20^, ^21^, ^22^, ^23^, ^24^, ^25^, ^26^, ^27^, ^28^, ^29^, ^30^, ^31^, ^32^, ^33^, ^34^, ^35^, ^36^, ^37^, ^38^, ^39^, ^40^, ^41^, ^42^, ^43^, ^44^, ^45^, ^46^, ^47^, ^48^	73.3%	86.7% Severe disease (S3) 13.3% Moderate disease (S2)	Initial FMT given followed by a 2^nd^ FMT after 1 week, followed by 1 short course of steroid therapy and monitoring for 3 months after the 2^nd^ FMT	Faecal samples from patients and donors pre-FMT, 1 week post-FMT and 4 months post-FMT were collected and stored for microbiota analysis by 16S rRNA sequencing
Chen *et al* (2020)^27^	47	Before vs after FMT treatment (+ comparison against the healthy donor profile)	**Treatment protocol** Single-donor FMT via colonic transendoscopic enteral tubing **FMT preparation** Single donor per patient	44.4 (±15.5)	57%	5.9 (±2.0) Total Mayo score	Total of 3 FMT treatments given every other day for 1 week	Molecular microbiological analyses were performed using faecal samples obtained from patients 1 day prior to FMT, and 4 and 12 weeks after FMT
Sood *et al* (2020)^28^	140	No active comparator	**Treatment protocol** Multi-session FMT via colonoscopy **FMT preparation** Single donor per patient	35 (±11)	62.36%	8.07 (±2.00) Mean Mayo Clinic score	FMT treatment at weeks 0, 2, 6, 10, 14, 18 and 22	Single-centre prospectively analysis patients with active UC treated with FMT. Predictive clinical biomarkers of response explored
Okahara *et al* (2020)^29^	92	Antibiotic treatment alone (n = 37)	**Treatment protocol** Antibiotic pre-treatment followed by FMT (n = 55) **FMT preparation** Single donor per patient	Mono-AFM arm – 42.5 (±14.7) A-FMT arm – 40.1 (±13.3)	Mono-AFM arm – 48.7% A-FMT arm – 69.1%	Mono-AFM arm – 1.8 (±0.8) Mayo Endoscopic score A-FMT arm – 1.9 (±0.7) Mayo Endoscopic score	Patients received antibiotic pre-treatment for 2 weeks prior to fresh FMT by colonoscopy	Clinical response was observed at 4 weeks post-treatment and maintenance response observed at 24 weeks post-treatment
Zhao *et al* (2021)^30^	116	No active comparator	**Treatment protocol** Variable infusions / treatment sets of FMT delivered via various routes – upper GI, lower GI or capsule. **FMT preparation** Unclear	Not reported	Not reported	Not reported	Unclear	Retrospective review of UC patients treated with FMT. Explored early recurrence – defined as an increase in Mayo score by ≥ 2 within one week of FMT. Predictive clinical biomarkers of response explored
Goyal *et al* (2018)^34^	21	Before vs after FMT treatment (+ comparison against the healthy donor profile)	**Treatment protocol** FMT delivery via faecal suspension into the distal duodenum or proximal jejnum, followed by a flush of normal saline and then delivery of faecal suspension into the terminal ileum and right colon **FMT preparation** Single donor per patient	12^8^, ^9^, ^10^, ^11^, ^12^, ^13^, ^14^, ^15^, ^16^, ^17^, ^18^, ^19^, ^20^, ^21^	57.1%	Mayo Endoscopic score 1 – 33.3% Mayo Endoscopic score 2 – 41.7% Mayo Endoscopic score 3 – 16.7%	Patients received 1 infusion	Patients treated with antibiotics (metronidazole/vancomycin) for 5 days starting 7 days before FMT Patients also took omeprazole (or equivalent) for 7 days starting 5 days prior to FMT All participants received 2-4mg of loperamide 2 hours prior to FMT Clinical response and adverse events were assessed at I week, 1 month, and 6 months after FMT
Uygun *et al* (2017)^38^	30	Before vs after FMT treatment	**Treatment protocol** Pre-treatment with bowel lavage followed by FMT via endoscopic infusion catheter **FMT preparation** Single donor per patient	34.6 (±10.3)	46.7%	Severe disease – 66.7% Moderate disease – 33.3%	Patients received 1 infusion	Fresh stool samples from the donors were collected Clinical remission and response rates were calculated for participants at week 12 post-FMT
Nishida *et al* (2016)^36^	41	Before vs after FMT treatment (+ comparison against the healthy donor profile)	**Treatment protocol** Single FMT infusion via colonoscopy **FMT preparation** Single donor per patient	39.6 (±16.9)	68.3%	5.6 (±2.4) Full Mayo score	Patients received 1 infusion	Primary end point – clinical response at 8 weeks
Gogokhia *et al* (2019)^39^	20	Before vs after FMT treatment (+ comparison against the healthy donor profile)	**Treatment protocol** Two-donor FMT via colonoscopy to the terminal ileum **FMT preparation** Unclear if multiple / pooled donors or single donor per patient				Patients received 1 infusion	Faecal samples collected pre-FMT, week 2 and week 4 post-FMT

⁎ consisting of ≥ 10 patients in FMT treated arms

† non-placebo controlled blinded randomised trials FMT-faecal microbiota transplantation, cFMT-capsulised faecal microbiota transplantation, dFMT-donor FMT, aFMT-autologous FMT, SCCAI-simple clinical colitis activity index.

### Changes in microbial diversity

Five RCTs reported on changes in alpha diversity following FMT as presented in Table 3.^7^^,^^9^, ^10^, ^11^^,^^13^ Three observed a significant increase in alpha diversity relative to baseline following FMT in all patients regardless of clinical response.^7^^,^^9^^,^^11^ The FOCUS study observed this change being more pronounced in patients who entered clinical remission compared to those who did not.^7^^,^^33^ In contrast the LOTUS study and the RCT by Costello and colleagues observed that the increase in alpha diversity following FMT was no longer significant when stratified by clinical response.^9^^,^^11^ In comparison, the TURN trial observed a significant increase in alpha diversity in both donor FMT and autologous FMT responders but not in non-responders.^10^^,^^31^ Amongst the non-randomised studies, only one study consisting demonstrated a significant increase in alpha diversity at post-FMT compared with pre-FMT, with this effect disappearing at 6 months.^34^ Non-significant trends reported including in increase in alpha diversity following FMT were observed in three non-randomised studies^22^^,^^24^^,^^27^ and one study showed a decrease in diversity with each sequential FMT treatment.^21^

**Table 3:** Studies characterising changes in microbial diversity and profiles following FMT for UC

Reference	Bioinformatic methodology	α + β Diversity (after FMT)		Taxonomic Changes (after FMT)		
		Responders	Non-responders	Responders	Non-responders	
Paramsothy *et al* (2019)^33^	**16S rRNA analysis** MOTHUR pipeline **Shotgun metagenomics** Filtering - DeconSeq, FastQC Analysis - SolexaQA, MetaPhlAn2, HUMAnN2.	In all patients α-diversity ↑ (Phylogenetic, richness and Shannon's diversity; P < 0.0001). β-diversity (multivariate dispersion) changed (P = 0.0001) following FMT, however these were more pronounced in patients entering remission		↑ faecal + mucosal species richness ↑ *Eubacterium hallii* (Firmicutes)*, Roseburia inulinivorans* (Firmicutes Lachnospiraceae)*, Eggerthella species* and *Ruminococcus bromii* (Firmicutes *Ruminococcus*) ↑ Firmicutes (*Oscillibacter* and *Clostridium XVIII*)	↓ faecal + mucosal species richness ↑ *Fusobacterium* (*Fusobacterium gonidiaformans*) (most consistent association), *Sutterella* (*Sutterella wadsworthensis*)*, Haemophilus, Escherichia, Megamonas, Clostridium XIVa, Prevotella* (*Prevotella copri*) *Dialister, Veillonella* and *Bilophila*	
Moayyedi *et al* (2015)^8^	**16S rRNA analysis** Analysis – Phyloseq R package and QIIME.	α-diversity not reported Significant change in β-diversity (Bray-Curtis dissimilarity) following FMT with no association with clinical response. (P = 0.02)		↑ Lachnospiraceae family and *Ruminococcus* in donor B (associated with successful FMT)	↑ *Escherichia* and *Streptococcus* in donor A	
Costello *et al* (2019)^9^	**16S rRNA analysis** Unspecified in-house and open source software. Differential abundance analysis lme4, mice, and glmmTMB R packages	↑ α-diversity (OTU analysis) in all patients following FMT with no association with clinical response. β-diversity not reported		↑ *Methanobrevibacter smithii,* Peptococcus niger (Firmicutes), Faecalicoccus pleomorphus (Firmicutes), Olsenella sp. (Actinobacteria), *Acidaminococcus intestini* (Firmicutes)*, Senegalimassilia anaerobia* (Actinobacteria)*, Prevotella copri* (Bacteroidetes)*, Clostridium methylpentosum* (Firmicutes)*, Alistipes indistinctus* (Bacteroidetes)*,* Slackia isoflavoniconvertens (Actinobacteria) and Odoribacter splanchnicus strain (Bacteroidetes) ↓ *Anaerostipes caccae, Gordonibacter pamelaeae* and C*lostridium aldenense* Abundance change in *Anaerofilum pentosovorans* (Firmicutes)*, Bacteroides coprophilus* (Bacteroidetes)*, Clostridium methylpentosum* (Firmicutes)*, Acidaminococcus intestini* (Firmicutes)*, Senegalimassilia anaerobia* (Actinobacteria)	Abundance change in *Fusicatenibacter saccharivorans* (Firmicutes) and *Paraprevotella xylaniphila* (Bacteroidetes)	
Rossen *et al* (2015)^10^	**16S rRNA analysis** USEARCH algorithms and unspecified independent classification techniques. Differential abundance analysis using Canoco5	↑ α-diversity (Shannon's index, P = 0.06 (FMT-D), P = 0.01 (FMT-A)) β-diversity shift (redundancy)	No change in diversity	↑ *Clostridium IV, XIVa* and *XVIII* (Firmicutes)(FMT-D responders) ↓ Bacteroidetes (FMT-D responders) ↑ *Bacilli,* Proteobacteria and Bacteroidetes (FMT-A responders)	None presented	
Crothers *et al* (2021)^13^	**16s rRNA analysis** QIIME2 pipeline	No change in α-diversity (Shannon Index)		Taxonomic data not presented		
Pai *et al* (2021)^12^	**16S rRNA analysis** Custom Perl scripts, Phyloseq R package and QIIME.	α-diversity not reported β-diversity (unspecified measure) changed in FMT arm (not significant) – no association with clinical response		*Alistipes spp.* and *Escherichia spp.* associated with achieving composite clinical outcome	None presented	
Haifer *et al* (2021)^11^	**16S rRNA analysis** MOTHUR pipeline	α diversity (richness) ↑ in all patients with changes seen in β-diversity (ANOSIM) following FMT. However, no change in α or β in relation to response or non-response.		Increase in Bacteroides OTU19 (100% similarity to *Bacteroides ovatus* and *Bacteroides xylanisolvens*)	Increase in Bacteroides OTU14 (100% similarity to *Bacteroides caccae* increase	
Tian *et al* (2019)^21^	**16S rRNA analysis** Full pipeline not described. Differential analysis using LEfSe.	α-diversity (Shannon index and Chao I index) and β-diversity (ANOVA) unchanged following FMT in all patients with no association with clinical response.		↑ Bacteroidetes*, Proteus, Prevotella, Phascolarctobacterium* and *Lactobacillus* (d1), *Clostridiaceae* (d2) ↓ Firmicutes*, Streptococcus*	↑ Bacteroidetes*,* Proteus ↓ Firmicutes*, Streptococcus*	
Li *et al* (2020)^22^	**16S rRNA analysis** Combination of MOTHUR, UPARSE and R	α-diversity ↑ (Shannon index and Chao I index) β-diversity (MDS) shift (trend) (both analogous to the donors). No separate data in responders		↑ *Holdemania* *Anaerostipes, Bifidobacterium, Clostridium IV* and *Odoribacter* (analogous to donors) *Eubacterium* and *Ruminococcus* (close to donors) Differences in relative abundance of *Eggerthella, Lactobacillus* and *Ruminococcus* positively correlated to efficacy (P < 0.05)	Notable difference in *Eubacterium* and *Ruminococcus* abundance compared with donors (P < 0.001)	
Leonardi *et al* (2020)^32^	**ITS1 analysis** BLAST with ITS1 database fllowed by QIIME v1.6 **Bacterial analysis** as per Paramsothy et al. (2019)^33^	↑ bacterial α-diversity (↑ *Candida* pre-FMT had ↑ α-diversity 8 weeks post-FMT) No change to mycobiota diversity. No association with clinical response		Reduction in abundance of *Candida* positively associated with clinical response	No change in relative abundance of *Candida*	
Kump *et al* (2018)^23^	**16S rRNA analysis** Combination of UCHIME, MOTHUR and QIIME v1.8	No change in α-diversity (richness) Significant change in β-diversity (unweighted UniFrac distance)		↑ *Akkermansia muciniphila* ↓ *Dialister*	No increase in *A. muciniphila*	
Jacob *et al* (2017)^24^	**16S rRNA analysis** USEARCH and UPARSE algorithms / pipelines	α-diversity ↑(OTUs P = 0.0049, Shannon index P = 0.069) Difference in β-diversity (Bray-Curtis dissimilarity) post-FMT (P < 0.034). No association with clinical response.		No taxonomic data presented		
Fang *et al* (2021)^25^	**16S rRNA analysis** Full pipeline not described. Differential analysis using LEfSe.	No difference in α-diversity (Kruskal–Wallis rank sum).		↑ Bacteroidetes and *Prevotella and* ↓ Proteobacteria and *Escherichia* post FMT. Association with clinical response data not presented.		
Cui *et al* (2015)^26^	**16S rRNA analysis** Not described.	Microbial analysis only performed on a subset of patients (n=4). ↑ α-diversity seen in 3 patients post FMT (Pearson correlation coefficient)		No taxonomic data presented		
Chen *et al* (2020)^27^	**16S rRNA analysis** UPARSE and QIIME v1.7	↑ α-diversity (Shannon index) week 4 but then ↓ at week 12 – no association with clinical response		↑ *F. Prausnitzii* (P < 0.05) – no association with clinical response		
Brezina *et al* (2021)^20^	**16S rRNA analysis** QIIME2 pipeline. Differential analysis using LEfSe.	α-diversity ↑ (Shannon entropy index)		↑ *Bacteroidales, Prevotellaceae, Veilllonellaceae* and *Desulfobacteria*	↑ *Staphylococcaceae, Lactobacillaceae* and *Bifidobacteriaceae*	
Fuentes *et al* (2017)^31^	**16S rRNA** USEARCH algorithms and unspecified independent classification techniques. Differential abundance analysis using Canoco5	Analysis of TURN patients		↑ *Clostridium XIVa* (*Anaerostipes caccae, Coprococcus eutactus* or *Eubacterium rectale* (similar levels to healthy donors)) ↓ *Enterococcus,* Proteobacteria Positive association to *Clostridium IV* (*F. prausnitzii*) and *XIVa* (*Eubacterium hallii, Roseburia intestinalis* and *Butyrivibria crossotus*)	↓ *Clostridium XIVa* (*Anaerostipes caccae, Coprococcus eutactus* or *Eubacterium rectale*) ↑ *Enterococcus,* Proteobacteria and *R. gnavus* (P = 0.014) Positive association with Bacteroidetes groups (*B. vulgatus* and *B. fragilis*)	
Goyal *et al* (2018)^34^	**16S rRNA analysis** QIIME pipeline. Differential analysis using LEfSe.	↑ α-diversity (OTU) Change in β-diversity (weighted UniFrac) - both seen 1-month post-FMT. No statistically significant difference in α-diversity seen at 6 months post-FMT	No significant increase in α-diversity (OTU) at 1- and 6-months post-FMT No change in β-diversity (weighted UniFrac) 1-month post-FMT	↑ *Lachnospiraceae* and ↓ *Enterobacteriaceae* at 1 week, 1 month and 6 months post-FMT		
Nishida *et al* (2016)^36^	**16S rRNA analysis** Full pipeline not described. Phyloseq R package for diversity analysis	No difference in α- and β-diversity (Bray-Curtis dissimilarity index) at week 8		No taxonomic data presented		
Gogokhia *et al* (2020)^39^	**Virome analysis** Filtering using BBMAP following by analysis usin VirMAP pipeline	Not reported		No change in relative abundance of Caudovirales bacteriophages 4 weeks post FMT		Increase in relative abundance of Caudovirales bacteriophages 4 weeks post FMT

FMT-faecal microbiota transplantation, OUT-Operational taxonomic units, QIIME-Quantitative Insights Into Microbial Ecology, LEfSe-Linear discriminant analysis Effect Size, MDS-Multidimensional scaling

Five RCTs reported on changes in beta diversity following FMT.^7^^,^^8^^,^^10^, ^11^, ^12^^,^^31^^,^^33^ Four observed a significant change in beta diversity following FMT in comparison to the placebo/inactive arm and relative to pre-FMT baseline.^7^^,^^8^^,^^10^^,^^11^^,^^31^^,^^33^ Both the FOCUS trial and the RCT by Moayyedi and colleagues demonstrated a significant difference in the gut microbial composition following FMT. Furthermore, they demonstrated the gut microbial profiles following FMT were more similar to donors regardless of clinical response with Moayyedi demonstrating that this similarity was only seen between FMT treated recipient and their respective donor. Similarly, the TURN trial demonstrated that the microbiota composition of responders in the donor FMT group shifted from overlap with non-responders at baseline to healthy donors following FMT.^31^ These microbial compositional shifts were not however observed in the patients treated with autologous FMT.

Five non-randomised studies measured changes to beta diversity in UC patients receiving FMT.^21^, ^22^, ^23^, ^24^^,^^35^ Of these three studies demonstrated a change in beta diversity following FMT relative to baseline community profiles.^23^^,^^24^ The study by Jacob *et al* and Goyal *et al* demonstrated that this shift in the beta diversity resulted in a greater similarity with the donor faecal microbiota.^24^^,^^34^ A similar donor-recipient similarly trend in beta diversity was observed by Li *et al* however no clear difference between responders and non-responders following FMT was seen.^22^

### Taxonomic changes

Six of the seven eligible placebo controlled RCTs reported on microbial taxonomic changes following FMT through analysis of stool 16S rRNA profiles as presented in Table 3.^7^, ^8^, ^9^, ^10^, ^11^, ^12^ In addition, the FOCUS trial performed stool metagenomic analysis and 16S rRNA on colonic mucosal biopsies collected at baseline and at the end of the FMT treatment period (week 8).^33^

### Changes associated with response to FMT

A significant increase in taxa belong to the Clostridia class (specifically XVIII) in responders to FMT were observed in four RCTs.^8^^,^^9^^,^^31^^,^^33^ Notably within this class and increase in taxa belonging to the families *Oscillospiraceae* (*Ruminococcus bromii, Anaerofilum pentosovorans, Clostridium methylpentosum*), *Lachnospiraceae* (*Roseburia inulinivorans, Eubacterium hallii) and Clostridiaceae* was observed in responders. Increases in taxa belonging to the Clostridia class were also reported in several of the non-randomised FMT studies. *Faecalibacterium prausnitzii* was reported to significantly increase in responders 4 weeks post-FMT relative to baseline.^27^ A significantly lower relative abundance of *Ruminococcus* and Eubacterium compared to healthy donors was reported in non-responders to FMT in a study by Li and colleagues with a non-significant increase in *Ruminococcus* in responders.^22^

Four studies reported a significant increase in taxa belonging to phylum Bacterioidetes following FMT in responders.^9^^,^^11^^,^^12^^,^^31^ Specifically, these included *Bacteroides coprophilus, Bacteroides OTU19* (100% similarity to *Bacteroides ovatus* and *Bacteroides xylanisolvens*) and *Alistipes spp.*

In addition to Clostridia and Bacteroidetes, a significant increase was reported in *Eggerthella* (Actinobacteria), *Senegalimassilia anaerobia* (Actinobacteria), *Acidaminococcus intestine* (Negativicutes) and *Escherichia* (Proteobacteria). Within the non-placebo controlled or non-randomised studies Brezina and colleagues demonstrated a significant increase in *Bacteroidales, Prevotellaceae, Veillonellaceae* and *Desulfobacteria* in responders.^20^ A significant increase in taxa belonging to the order *Bacteroidales* and *Verrucomicrobiales* and class *Coriobacteriia* was noted in responders compared to non-responders in another study*.*^22^ The non-randomised paediatric FMT study by Goyal *et al* demonstrated a significant decrease in *Enterobacteriaceae* and an increase in *Lachnospiraceae* following FMT, however this difference was not significant when sub-grouped by response.^34^

Analysis of the gut mycobiome, as part of a post-hoc analysis of the FOCUS trial noted that decreased *Candida* abundance post-FMT was indicative of clinical response.^32^ The LOTUS study in contrast did not report any changes in alpha or beta diversity metrics of the mycobiome upon disease flare.^11^

### Changes associated with lack of response to FMT

Changes in microbial taxa associated with lack of response to FMT were reported by four RCTs.^8^^,^^9^^,^^11^^,^^33^ These included a significant increase in species belonging to phylum Fusobacteria (*Fusobacterium gonidiaformans*), phylum Proteobacteria (*Bilophila, Haemophilus, Escherichia, Sutterella wadsworthensis*) and family *Prevotellaceae* (*Paraprevotella xylaniphila, Prevotella copri).* In addition, a significant increase in *Dialister, Veillonella, Megamonas, Fusicatenibacter saccharivorans, Clostridium XIVa* and *Bacteroides OTU14* (100% similarity to *Bacteroides caccae*) was observed in non-responders. Responders in the LOTUS trial who developed a disease flare on FMT withdrawal had an enrichment of *Streptococcus OTU45* (100% similarity to *Streptococcus parasanguinis* and other phylogenetically related species) along with depletion of *Blautia OTU35* (100% similarity to *Blautia faecis*).^11^ No clear alpha diversity change was however noted. Within the non-placebo controlled or non-randomised studies Brezina and colleagues demonstrated that *Staphylococcaceae, Lactobacillaceae* and *Bifidobacteriaceae* were significantly higher in non-responders.^20^

### Metabolomic analysis

Two RCTs^9^^,^^33^ analysed changes in microbial metabolites following FMT treatment. The FOCUS trial identified 97 metabolites that were different between baseline and following FMT treatment regardless of clinical response.^33^ Of these metabolites, N-acetylmuramate, xanthine, 2-deoxyinosine, ribothymidine and X- 17009 (unnamed biochemical) were significantly increase post-FMT but were not altered by placebo. The trial reported significant differences in global metabolomic profiles following FMT in clinical responders in comparison to baseline, after placebo and after FMT in clinical non-responders. Specifically, 228 metabolites differentiated between positive and negative outcomes following FMT of which 33 of these were different in patients achieving clinical response. Metabolites such belonging to benzoate degradation, glycerophospholipid metabolism, secondary bile acid biosynthesis, ppGpp biosynthesis and biosynthesis of ansamycins pathways were associated with positive outcomes following FMT. In contrast metabolites associated with heme and lysine metabolic pathways were associated with a negative outcome after FMT. Faecal metabolome analysis in the Costello study that was specifically targeted to short chain fatty acid levels reported no significant differences from baseline in stool concentrations of butyrate, acetate, propionate, iso-butyrate, valerate, iso-valerate and caproate following FMT regardless of clinical response or treatment arm (donor versus autologous).^9^

Whilst TURN trial did not report changes in faecal metabolic profiles they performed functional predictive analysis using PICRUSt and qPCR.^31^ Microbiota of non-responders in this study had a significantly lower butyrate production capacity, reflected by the butyrate-acetoacetate CoA transferase and *ButCoA* gene copies, compared with donors and responders. *ButCoA* levels were increased by 6.7-fold in responders, especially those who remained in remission at ⩾1-year FU. A non-randomised study that used similar predictive functional analysis gut microbiota reported on significant differences in pathways of pyruvate metabolism, sulfur metabolism, pantothenate and CoA biosynthesis, glyoxylate and dicarboxylate metabolism, synthesis and degradation of ketone bodies and other transporters were between donor, pre- and post-FMT groups.^25^

### Donor characteristics association with clinical response

Two RCTs that explored donor recipient association demonstrated that microbial profiles of recipients were significantly more similar to their respective donors following FMT compared to controls as presented in Table 4.^8^^,^^31^ Notably the study by Moayyedi and colleagues noted that one particular donor, ‘Donor B’, was associated with greater success rate) in their respective recipients with a non-significant trend for faecal microbiota from responders having greater similarly to donor B than non-responders.^8^

**Table 4:** Data summary table of the relationship between patients’ and donors’ microbiota post-FMT

Reference	Donor Relationship (after FMT)	
	Responders	Non-responders
Paramsothy *et al* (2019)^33^	↑ homogeneity in taxonomic profiles to a level seen in donors Donor batches with ↑ *Bacteroides* OTU187 (*Bacteroides fragilis* and *Bacteroides finegoldii*)	Donor batches with ↑ *Clostridium* XIVA and association with *Bacteroides uniformis, Bacteroides coprocola* and *Streptococcus* OTU56
Moayyedi *et al* (2015)^8^	↑ microbiota similarity to donor B (enrichment of *Lachnospiraceae* and *Ruminococcus*)	↓ microbiota similarity to donor B
Rossen *et al* (2015)^10^	• Microbiota composition overlap with healthy donors (FMT-D) characterised by ↑ Clostridium clusters IV, XIVa, XVIII and ↓ Bacteroidetes • Microbiota composition shift away from non-responders (FMT-A (different direction to FMT-D responders)) characterised by ↑ Bacilli, Proteobacteria and Bacteroidetes ↑ similarity index to corresponding donors ↑ similarity to donors which they received faeces from (P = 0.02)	↓ similarity index to corresponding donors ↓ similarity to donors which they received faeces from (P = 0.02)
Haifer *et al* (2021)^11^	Donor 1 (favourable donor) had a significantly higher bacterial diversity driven by higher species evenness with compositional differences largely related to differences in relative abundances of Bacteroidetes taxa	Not reported
Jacob *et al* (2017)^24^	↑ Similarity with donor FMT samples Donors achieving clinical remission clustered together	Not reported
Chen *et al* (2020)^27^	Abundance of *F. prausnitzii* ↑ towards levels similar to those of donors	Not reported
Li *et al* (2020)^22^	↓ Dissimilarity between patients and donors (α + β diversities analogous to donors)	↓ Dissimilarity between patients and donors (α + β diversities analogous to donors)
Fuentes *et al* (2017)^31^	↑ Similarity to donors (FMT-D) (P = 0.02) Trend of ↑ similarity to donors (patients with sustained remission) (P = 0.1) No significant differences in similarity values of FMT-A patients	↓ Similarity to donors (FMT-D) (P = 0.02) Trend of ↓ similarity to donors (relapsers) (P = 0.1) Donor batches associated with Proteobacteria (*E. coli* and *Aeromonas*) and ↑ abundance of *Ruminococcus gnavus* No significant differences in similarity values of FMT-A patients
Kump *et al* (2018)^23^	All recipients’ microbiotas, regardless of response, shifted towards the respective donor microbiota	All recipients’ microbiotas, regardless of response, shifted towards the respective donor microbiota
Shabat *et al* (2021)^19^	UCED preconditioning of donors led to reduction of alpha diversity of donor stool with numerically higher remission rates compared with FMT alone (or UCED and FMT).	
Okahara *et al* (2020)^29^	↑ Cumulative non-relapse rate in sibling FMT than parent-child FMT Donor Bacteroidetes species (*Bacteroides uniformis* and *Parabacteroides distasonis* and *Bacteroides dorei*) persisted in patients with no UC recurrence after 24 months ↑ Similarity of 10 Bacteroidetes species to donor levels	↓ Cumulative non-relapse rate in ≥11-year difference group that 0-10-year difference group

FMT-faecal microbiota transplantation, FMT-D-donor faecal microbiota transplantation, FMT-A-autologous faecal microbiota transplantation, UCED-Ulcerative colitis exclusion diet

Four RCTS reported on the association of clinical response with taxonomic characteristics in donor stool with inconsistent findings.^8^^,^^11^^,^^31^^,^^33^ Abundance of specific taxa belonging to Bacteroidetes phylum within donor stool and correlation with a favourable clinical response have been observed in both the FOCUS and LOTUS clinical trials. As the FOCUS trial used pooled FMT, specific donor-recipient relationships could not be explored. Effective donor batches leading to >50% remission in patients contained a higher abundance of *Bacteroides* OTU187, specifically *Bacteroides fragilis* and *Bacteroides finegoldii*, whilst ineffective batches were associated with *Clostridium* XIVA. There was also a non-significant trend towards an association between ineffective batches and the taxa *Bacteroides uniformis, Bacteroides coprocola, Sutterella Wadsworthenesis* and *Streptococcus* OTU56. The LOTUS study manufactured oral lyophilised FMT capsules from two separate donors. They demonstrated that the donor with a significantly higher bacterial diversity (greater species evenness) with significant differences in relative abundances of Bacteroidetes taxa was associated with a favourable clinical response. Higher taxonomic classification was however not provided in the study. An open label non-randomised study demonstrated that clinical response was significantly greater donors with a higher abundance of faecal *Bifidobacterium, Lactobacillales* and *Clostridium* clusters IV and XI.^36^ No significant difference in donor-recipient gut microbial similarity was observed between responders and non-responders.

Moayyedi and collegues noted that Donor B had enrichment of *Lachnospiraceae* and the genera *Ruminococcus*. In contrast, the TURN study observed a greater abundance of *Ruminococcus gnavus* in donors of patients who relapsed compared with donors of patients who achieved sustained remission. However, post-hoc analysis of the TURN study with at least one year follow up of patients in this trial observed that donor faecal samples consisting of *E. coli* and *Aeromonas* were positively associated with patients who relapsed.^31^

Donor (and recipient) faecal microbiome optimisation prior to stool collection and FMT administration was explored in the CRAFT UC study.^19^ A specific diet named UC exclusion diet (UCED) was administered as part of this study and comprised mandatory foods such as certain fruits and vegetables, prescribed amounts of chicken and eggs and certain foods that were restricted with the aim of decreasing exposure to sulphated amino acids, total protein, heme, saturated fat and food additives. Donor and recipient dietary conditioning UCED was attempted with patients randomised to either Group 1 - standard low intensity FMT followed by standard diet, Group 2 – low intensity FMT from donors pre-conditioned with UCED and post FMT recipient conditioning with UCED or Group 3 - UCED alone. Numerically higher, but not statistically significant clinical remission rates and mucosal healing in Group 3 (UCED alone) compared to the FMT arms (Groups 1 and 2). The authors showed that the UCED diet preconditioning of donors reduced the alpha diversity of donor stool microbiota rather than an anticipated increase. Recipient microbiome data or donor-recipient response was not presented as part of the study.

### Baseline predictors of response

#### Clinical predictors

Baseline clinical predictors were reported in three RCTs and two non-randomised studies. Using demographic information obtained from baseline questionnaires, Moayyedi *et al* reported a trend towards patients receiving immunosuppressant therapy at baseline acquiring a greater benefit from FMT.^8^ Additionally, the authors found that patients were statistically significantly far more likely to respond to FMT if they had received a recent diagnosis of UC (defined as ≤1 year). In contrast the FOCUS trial observed an inverse relationship between endoscopic severity and the primary outcome however this was no longer seen when controlled for other factors.^7^ Correlation with clinical response was also noted with age but directionality was not reported. No relation was however observed between the primary outcome and anatomical disease extent, smoking status, disease duration, any concomitant immunosuppressive (steroids, biologics, immunomodulatory) use. Similarly, the RCT by Costello *et al* did not observe any interactions between age at diagnosis/randomisation, disease duration/distribution, gender, non-steroid based medication use or macronutrient intake with a change in total Mayo score following donor FMT.^9^ Use of oral steroids at randomisation was however associated with a greater reduction in total Mayo score.

Amongst the non-randomised studies, in a single-centre prospectively study of open label FMT statistically significant association between moderate disease severity (Mayo score 6-9) and remission in UC patients, along with endoscopic Mayo score 2.^28^ In addition, the authors noted that severe disease (Mayo score ≥10) and endoscopic Mayo score 3 were both significantly correlated with FMT failure. A previous study by the same authors reported that patients treated earlier on in the disease course or those with mild disease had higher rates of clinical remission.^37^ They noticed that in biologic-experienced patients, endoscopic Mayo score 2 was a predictor of response whereas in biologic-naïve patients younger age, moderate disease severity, shorter disease duration and endoscopic Mayo score 2 were all significantly predictive of a positive outcome. They described young age as a baseline factor which determined participants’ response with patients under 40 years demonstrating greater rates of remission. In a univariate analysis performed in an uncontrolled study that consisted 11 disease recurrences in 116 UC patients with active disease reported significant associations with a baseline high Mayo score, recent use of steroids to induce remission, low serum albumin, and peripheral blood lymphocyte deficiency were associated with a higher recurrence rate following FMT.^30^ These are however recognised factors associated with unfavourable disease outcomes irrespective of treatment. No association with disease extent was observed and disease duration was not explored. Two other non-randomised studies did not demonstrate any differences in clinical characteristics between responders and non-responders.^36^^,^^38^

#### Microbial predictors

Analysis of potential baseline microbial predictors of response in the FOCUS trial found that patients who achieved the primary outcome tended to have higher faecal species richness at baseline compared with patients not achieving the primary outcome.^33^ They also observed a similar non-significant trend in the mucosal microbiome in which a higher baseline species richness as well as an increased abundance of specific species of Bacteroides (*B. fragilis* and *B. finegoldii*) with was associated with a positive therapeutic outcome(33). Gut mycobiome analysis of the FOCUS trial observed that a greater abundance of *Candida* pre-FMT was associated with a clinical response (and increased bacterial diversity post-FMT).^32^ An open label study of 20 patients with active UC observed that FMT responders had a lower relative abundance of *Caudovirales* bacteriophages at baseline compared to non-responders. The relative abundance of *Caudovirales* in non-responders appeared to increase after FMT while no change was observed in responders.^39^

Patients receiving autologous FMT in the TURN trial had a greater likelihood of response to treatment if they possessed baseline microbiota profiles more similar to donor samples or to patients in sustained remission following donor FMT. Differences in baseline microbiota profiles between responders and non-responders was however not found to be a predictor of response for patients receiving donor FMT.^31^ Higher levels of Bacteroidetes, particularly *B. vulgatus*, and *Prevotella* in non-responders at baseline were associated with relapse at the 1 year follow up. A non-randomised study of FMT in paediatric patients with UC observed that the abundance of Fusobacterium was significantly greater at baseline in non-responders compared to responders.^34^

#### Metabolomic predictors

Potential baseline metabolomic predictors of response was only reported as part of the FOCUS study.^33^ Fifteen metabolites were identified-N-methylphenylalanine, N-acetylarginine, caproate, lignoceroyl ethanolamide, biotin were associated with an increased positive clinical outcome whilst the metabolites 5-aminovalerate, oleoyl-arachidonoyl-glycerol, linoleoyl-arachidonoyl-glycerol, linoleoyl-arachidonoyl-glycerol, sphingomyelin, sphingomyelin, gulonate and heme were identified as being associated with increased negative outcome.

## Discussion

**Figure 2:**
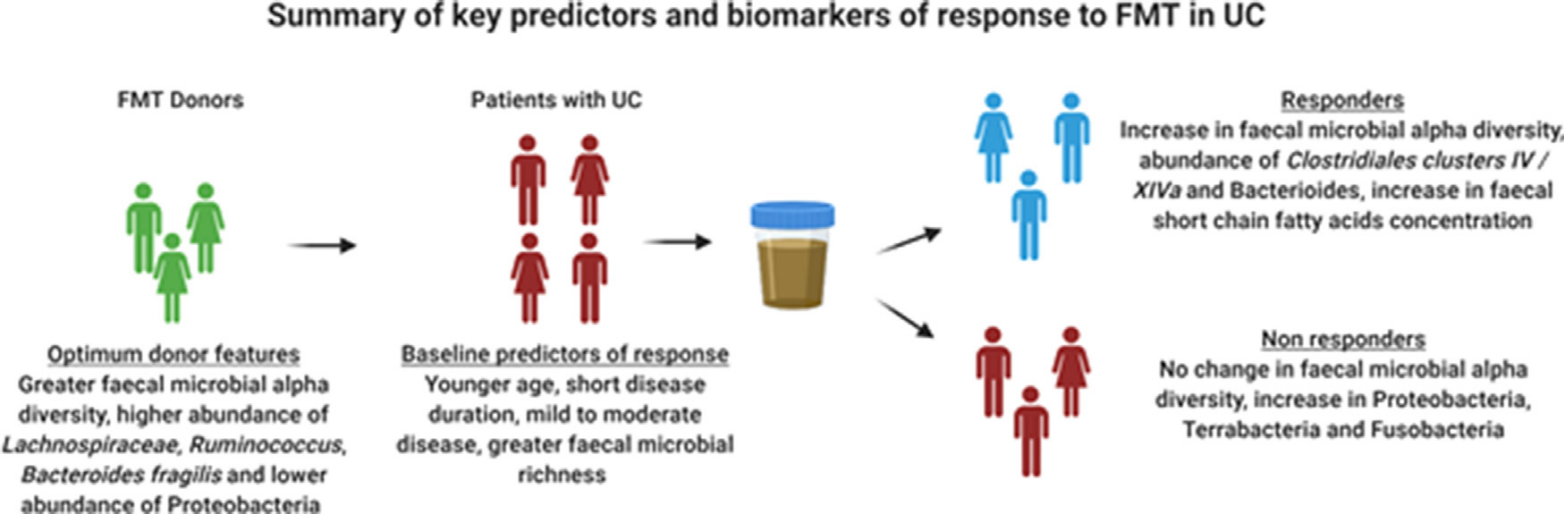
Summary of key predictors and biomarkers of response to FMT in UC. FMT, faecal microbiota transplantation, UC, ulcerative colitis.

This systematic review outlines potential donor and recipient clinical and microbial biomarkers that predict and denote clinical response to FMT in patients with UC. Examination of 7 double blind placebo controlled RCTs and 12 non-randomised studies in FMT in UC identified specific consistent findings in gut microbial profiles that correlate with a favourable clinical response along with clinical and microbial profiles that have the potential of predicting response to FMT (summarised in Figure 2).

Following FMT, the overall trends of biomarkers discovered in responding patients’ microbiota communities were (a) an increase in bacterial diversity (alpha and beta), (b) increases in Firmicutes and Bacteroidetes along with key taxa belonging to these phyla, and (c) recipient microbial profiles with increased similarity to donor profiles. Responders of FMT had microbial profiles more similar to that of their donors, possibly suggesting that the donor microbiota composition profile may be used as a potential microbial treatment target for individualisation of FMT treatment regimens. At a taxonomic level, studies have consistently demonstrated an increase in abundance of *Clostridium* clusters IV and XIVa (members of the Firmicutes phylum), post-FMT this is associated with a favourable clinical response. These include the *Lachnospiraceae* and *Ruminococcaceae* families and are likely to induce this response through immune regulation of colonic inflammatory pathways.^40^^,^^41^ SCFAs are the product of bacterial fermentation of polysaccharide, oligosaccharide and particular amino acids which are non-digestable by the host.^42^ Producers of SCFAs specifically *Clostridium* clusters have a crucial role in maintaining intestinal function.^43^ SCFAs have been shown to induce the differentiation of naïve CD4 T cells into immunosuppressive, anti-inflammatory IL-10-producing regulatory T cells.^41^^,^^44^ Consistently SCFA synthesis, and the presence of components contributing to this synthesis, appears to be a metabolomic biomarker of response post-FMT.^25^^,^^31^^,^^33^^,^^45^ For instance, observed gene copy levels of *ButCoA* were increased in those patients who received successful FMT therapy whilst the microbial capacity for butyrate production of the microbiota decreased in patients lacking a response to FMT.^31^ The FOCUS study also identified increased levels of heme and lipopolysaccharide biosynthesis at both baseline and post-FMT as potential biomarkers associated with a negative outcome.^33^ Not only do various bacterial pathogens produce heme, but it is also a vital source of iron required for their survival with murine studies suggesting its role in colonic inflammation.^46^

Certain baseline recipient characteristics were found to be important factors in determining a favourable outcome. FMT recipients with younger age, less severe and less extensive disease and potentially shorter duration of UC (< 1 year) have been shown to associated with a greater likelihood of response. These predictive baseline factors are not too different to that of biological/small molecule therapies in UC. Whilst other biomarkers were identified in this systematic review such as the prior or concurrent immunosuppressant use in predicting response, further research is needed to corroborate these findings.^8^ There is some evidence to suggest that patients with higher faecal microbial richness at baseline, greater abundance of *Candida,* lower abundance of *Caudovirales* and a microbial composition closer to donors at baseline are more likely to have a favourable response.^31^, ^32^, ^33^^,^^47^ It is plausible that the dysbiosis seen in patients with a recent UC diagnosis as well as a relatively lower degree of microbial aberrancy is more easily manipulated with FMT resulting in a greater likelihood of successful and sustained donor microbiome engraftment and clinical response.

Only the FOCUS study reported on baseline metabolomic predictors of response, with the findings of the RCT provide significant insight into the bacterial metabolites which give a higher likelihood of achieving a positive FMT outcome.^33^ One of the most notable metabolites was biotin (vitamin B7), with diet and synthesis by commensal microbiota in the gut being its primary source in humans.^48^ Biotin results in the downregulation of the NF-κB gene thereby restricting release of various pro-inflammatory cytokines in the gut epithelium.^49^

Greater microbial richness in donor stool was associated with an increased rate of clinical response in patients with active UC.^11^^,^^23^^,^^47^ Engraftment of donor-derived microbiota ameliorates UC symptoms through either replenishing bacterial species whose abundance is decreased prior to treatment or, providing bacteria which create an unfavourable environment for disease-associated bacteria so as to repress their growth.^50^ Having a high bacterial species richness, therefore, may increase the chances that certain bacterial strains engraft in the gut of the recipient and become permanent members of their microbiota community.^51^ Along with increased bacterial richness, specific taxa were identified in donor stool associated with remission, whilst others were found in those associated with treatment failure. Donor stool which included high abundances of *Bacteroides* OTU187 in addition to the families *Lachnospiraceae* and *Ruminococcaceae* were more likely to induce a response in recipients, whereas the presence of *Clostridium* XIVA was seen in ineffective batches.^8^^,^^11^^,^^33^ The TURN study in contrast observed a greater abundance of *Ruminococcus gnavus* in donors of patients who relapse. However, it is important to note that the microbial profiles of donors were similar to the baseline profiles of the UC patients in this study. Preselecting donors based on a richer microbial diversity and greater abundances of SCFA producing bacteria or pooling FMT from donors to control for variability in donor microbial diversity. Pooling FMT is, however, no longer practical as it presents major challenges with ‘look back’ exercises and root cause analysis in cases of FMT related adverse events. One option would be to pre-condition donors with a diet that is associated with increasing microbial diversity. The CRAFT UC study attempted this with preconditioning donors with a designer diet (UCED) that consisted of dietary exclusion of specific components such as saturated fat and food additives that are thought to contribute to an immune mediated inflammatory response.^19^ Paradoxically the UCED diet resulted in a reduction in donor microbial richness and may have potentially contributed to the unfavourable outcomes seen with donor pre-conditioned FMT. Nevertheless, optimum microbiome-based donor selection as well as pre-conditioning with a diet that is associated with increasing gut microbial diversity are likely to play an important role enhancing response with FMT.^52^^,^^53^

The findings of this systematic review highlight the possibility of enhancing a sustained response to FMT through biomarker-based selection and optimisation of donors and patients before and during the treatment with FMT. Utilising precision medicine, would facilitate an individualised, biomarker driven ‘treat to microbiome/metabolome’ target approach with FMT in UC early in the disease. After the pre-defined clinical target is reached, the need for further FMT is tracked based on loss of this specified microbiome target. Studies are now needed to help define these targets with leading candidates that include alpha diversity, specific faecal SCFA producing strains such as Clostridiales and faecal butyrate levels. There are a few limitations of this systematic review. The heterogeneity of the study designs that include mode and frequency of FMT administration, the use of a single or pooled donor approaches, variable placebo and active comparators and differences in microbial analytical strategies may make interpretation in the context of a systematic review challenging. However, the reproducibility and consistency of several of the findings reported in this review, in addition to biological plausibility, does bring a level of confidence. We excluded studies with less than ten (FMT treated) participants for quality control. None of these excluded studies had detailed exploratory mechanistic data that would have significantly influenced the findings in the review.

To conclude, there is evidence of existing predictive biomarkers for the treatment of UC with FMT, the most well-defined of these being microbial indicators. Despite the exponential growth in research into FMT over recent years, the mechanistic understanding on the basis of this treatment is poor. It also remains unclear if alterations to the microbiota occur to certain pre-existing immunomodulatory bacterial strains that are enriched post-FMT, or if they are solely donor derived and engrafted after treatment. It is clear however, that the gut microbiota is fast becoming a pivotal therapeutic target which holds considerable potential.

### Contributors

NPR and MNQ performed the search and data extraction. NPR wrote the first draft with critical feedback and edits from MNQ. All authors (NPR, WS, CQ, CT, RDH, NS, ADB, THI, MNQ) provided feedback and approved the final version of the draft.

## Declaration of interests

All authors declare no relevant conflict of interests.

## Appendix Supplementary materials

Image, application 1
