# Supplementary material for: Systematic review of donor and recipient predictive biomarkers of response to faecal microbiota transplantation in patients with ulcerative colitis
Source: eBioMedicine. 2022 May 31;81:104088. doi: 10.1016/j.ebiom.2022.104088 (PMC9163485; doi:10.1016/j.ebiom.2022.104088)
Supplement: Supplementary file 1 [file mmc1.docx]

**Supplementary Table 1. Systematic review database search terms**

| **Medline Search Strategy** | **Embase Search Strategy** |
| --- | --- |
| 1. inflammatory bowel disease/ 2. inflammatory bowel disease*.ti,ab. 3. IBD.ti,ab. 4. ulcerative colitis/ 5. ulcerative colitis.ti,ab. 6. UC.ti,ab. 7. fecal microbiota transplantation/ 8. FMT.ti,ab. 9. ((fecal or faecal or feces or faeces or stool or microbiota) adj2 (transplant* or infus* or transfuse* or implant* or instil* or donat* or donor or reconstitut* or therap* or bacteriotherapy or capsul* or encapsule*)).ti,ab. 10. or/1-6 11. or/7-9 12. 10 AND 11 | 1. exp inflammatory bowel disease/ or exp ulcerative colitis/ 2. (inflammatory bowel disease* OR IBD OR ulcerative colitis OR UC).ti,ab. 3. (((fecal or faecal or feces or faeces or stool or microbiota) adj2 (transplant* or infus* or transfus* or implant* or instil* or donat* or donor* or reconstitut* or therap* or bacteriotherapy or capsul* or encapsul*)) OR (fecal microbiota transplantation OR FMT)).ti,ab. 4. 1 OR 2 5. 3 AND 4 |

FMT-faecal microbiota transplantation, IBD-Inflammatory bowel disease, UC-ulcerative colitis

**Supplementary Table 2. RCT risk of bias table**

| **Reference** | **Random sequence generation** | **Allocation concealment** | **Blinding of participants and personnel** | **Blinding of outcome assessment** | **Incomplete outcome data** | **Selection reporting** |
| --- | --- | --- | --- | --- | --- | --- |
| Paramsothy *et al* (2019) (33) | Low risk | Low risk | Low risk | Some concerns | Low risk | Low risk |
| Moayyedi *et al* (2015) (7) | Low risk | Some concerns | Some concerns | Some concerns | High risk | Low risk |
| Costello *et al* (2019) (8) | Low risk | Some concerns | Low risk | Some concerns | Low risk | Low risk |
| Rossen *et al* (2015) (14) | Low risk | Low risk | Low risk | High risk | Low risk | Low risk |

RCT, randomised controlled trial.

**Supplementary Table 3 – Non-randomised studies risk of bias table**

| **Reference** | **NOS1**  Representativeness of the exposed cohort to average UC patient | **NOS2**  Similarity of exposed and control cohort populations | **NOS3**  Confirmation of FMT exposure | **NOS4**  Evidence outcome of interest (clinical remission/  response) was not present at start of study | **NOS5**  Study controls for disease severity | **NOS6**  Study controls for disease extent, duration or concomitant medications | **NOS7**  Assessment of outcome | **NOS8**  Adequate follow-up for outcomes of interest (1 month) | **NOS9**  Adequacy of follow up of cohorts | **NOS Total** |
| --- | --- | --- | --- | --- | --- | --- | --- | --- | --- | --- |
| Tian *et al* (2019) (16) | 0 | 0 | 1 | 1 | 0 | 0 | 1 | 0 | 1 | 4 |
| Nusbaum *et al* (2018) (20) | 1 | 0 | 1 | 1 | 0 | 0 | 1 | 1 | 1 | 6 |
| Li *et al* (2020) (21) | 1 | 0 | 1 | 1 | 0 | 0 | 1 | 1 | 0 | 5 |
| Kump *et al* (2013) (22) | 0 | 0 | 1 | 1 | 0 | 0 | 1 | 1 | 1 | 5 |
| Jacob *et al* (2018) (24) | 1 | 0 | 1 | 1 | 0 | 0 | 1 | 1 | 1 | 6 |
